# Aroylhydrazone Schiff Base Derived Cu(II) and V(V) Complexes: Efficient Catalysts towards Neat Microwave-Assisted Oxidation of Alcohols

**DOI:** 10.3390/ijms21082832

**Published:** 2020-04-18

**Authors:** Manas Sutradhar, Tannistha Roy Barman, Armando J. L. Pombeiro, Luísa M. D. R. S. Martins

**Affiliations:** Centro de Química Estrutural and Departamento de Engenharia Química, Instituto Superior Técnico, Universidade de Lisboa, Av. Rovisco Pais, 1049-001 Lisboa, Portugal; roybarman@tecnico.ulisboa.pt (T.R.B.); pombeiro@tecnico.ulisboa.pt (A.J.L.P.)

**Keywords:** Cu(II) complex, Schiff base, aroylhydrazone, X-ray structure, alcohol oxidation, microwave

## Abstract

A new hexa-nuclear Cu(II) complex [Cu_3_(μ_2_-1κ*NO^2^*,2κ*NO^2^*-L)(μ-Cl)_2_(Cl)(MeOH)(DMF)_2_]_2_ (**1**), where H_4_L = *N′^1^,N′^2^*-bis(2-hydroxybenzylidene)oxalohydrazide, was synthesized and fully characterized by IR spectroscopy, ESI-MS, elemental analysis, and single crystal X-ray diffraction. Complex **1** and the dinuclear oxidovanadium(V) one [{VO(OEt)(EtOH)}_2_(1κ*NO^2^*,2κ*NO^2^*-L)]·2H_2_O (**2**) were used as catalyst precursors for the neat oxidation of primary (cinnamyl alcohol) and secondary (1-phenyl ethanol, benzhydrol) benzyl alcohols and of the secondary aliphatic alcohol cyclohexanol, under microwave irradiation using *tert*-butyl hydroperoxide (TBHP) as oxidant. Oxidations proceed via radical mechanisms. The copper(II) compound **1** exhibited higher catalytic activity than the vanadium(V) complex **2** for all the tested alcohol substrates. The highest conversion was found for 1-phenylethanol, yielding 95.3% of acetophenone in the presence of **1** and in solvent and promoter-free conditions. This new Cu(II) complex was found to exhibit higher activity under milder reaction conditions than the reported aroylhydrazone Cu(II) analogues.

## 1. Introduction

Schiff base ligands play a key role in the progress of coordination chemistry due to their relevance in several interdisciplinary research fields. Aroylhydrazones, resulting from the condensation of an acid hydrazide and a carbonyl compound, are considered important Schiff base ligands able to stabilize transition metals with various oxidation numbers and different geometries [1,2,3,4,5]. Moreover, significant advances in the coordination chemistry of aroylhydrazones have been reported in view of the several important applications of their metal complexes, namely in biological systems, molecular magnetism, or catalytic processes [3,4,5,6,7,8,9,10,11,12,13,14]. The interesting feature of aroylhydrazone ligands is that they exhibit facile *keto*–*eno*l tautomerism in solution and modulate different kinds of coordination modes in transition-metal complexes [1,2,3,4,5,6,7,8,9,10,11,12,13,14]. 

From the knowledge that metal complexes bearing aroylhydrazone ligands exhibit high potential to act as efficient oxidation catalysts due to their stability towards oxidation [15,16], and in pursuit of our interest in developing sustainable catalytic alcohol oxidation processes [1,3,5,11,14,17,18], we have now chosen the bio-inspired earth-abundant copper metal to synthesize a new hexa-nuclear Cu(II) complex derived from *N′^1^,N′^2^*-bis(2-hydroxybenzylidene)oxalohydrazide [19,20,21,22], expanding the scope of multinuclear complexes synthesis. We also aimed to compare the catalytic properties of the new aroylhydrazone Cu(II) complex with a previously reported aroylhydrazone oxidovanadium(V) complex that proved its efficiency as pre-catalyst towards the peroxidative oxidation of gaseous and liquid alkanes [20] but was not applied for other substrates. Thus, herein we focused on the selective oxidation of alcohols to the corresponding ketones or aldehydes [23,24,25,26], which are important building blocks for the synthesis of many added-value organic compounds [27,28,29,30] and have applications in fields such as pharmaceuticals, agrochemicals, fragrances, and polymers [31,32]. Both complexes were tested as catalyst precursors towards the neat oxidation of primary (cinnamyl alcohol) or secondary (1-phenyl ethanol, benzhydrol) benzyl alcohols, as well as of the secondary aliphatic alcohol cyclohexanol using *tert*-butyl hydroperoxide (TBHP) as oxidizing agent under microwave (MW) irradiation, a relevant and simple energy-saving technique [14,33,34,35].

## 2. Results and Discussion

### 2.1. Synthesis and Structural Characterization

**Scheme 1:**
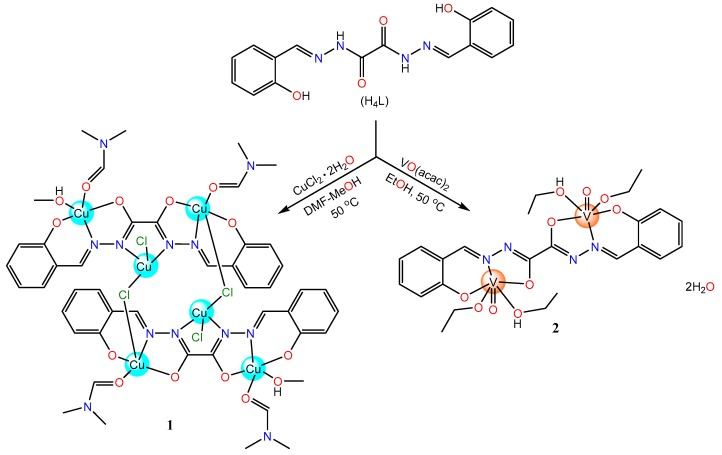
Synthesis of [Cu_3_(μ_2_-1κ*NO^2^*,2κ*NO^2^*-L)(μ-Cl)_2_(Cl)(MeOH)(DMF)_2_]_2_ (**1**) and [{VO(OEt)(EtOH)}_2_(1κ*NO^2^*,2κ*NO^2^*-L)]·2H_2_O (**2**).

The aroylhydrazone Schiff base *N′^1^,N′^2^*-bis(2-hydroxybenzylidene)oxalohydrazide (H_4_L) [19,20,21,22] was used to synthesize the hexa-nuclear Cu(II) complex [Cu_3_(μ_2_-1κ*NO^2^*,2κ*NO^2^*-L)(μ-Cl)_2_(Cl)(MeOH)(DMF)_2_]_2_ (**1**) and the dinuclear oxidovanadium(V) complex [{VO(OEt)(EtOH)}_2_(1κ*NO^2^*,2κ*NO^2^*-L)]·2H_2_O (**2**) (Scheme 1). Compound **1** is newly synthesized using CuCl_2_·2H_2_O and H_4_L in DMF–methanol medium. Compound **2** was obtained by a literature method [20]. Hosseini-Monfared et al. reported a similar binuclear oxidovanadium(V) complex derived from H_4_L where a methoxido ligand and a methanol molecule, instead of ethoxido and ethanol, respectively, are coordinated to the vanadium center [21]. They studied the catalytic activity of the complex towards the peroxidative oxidation of cyclooctene. A binuclear dioxidovanadium(V) complex of the same ligand was also found in the literature [19]. In both complexes **1** and **2,** the *N′^1^,N′^2^*-bis(2-hydroxybenzylidene)oxalohydrazide acts as a tetra-negative chelating ligand (L^4–^) with two tridentate *O*^−^*,N,O*^−^ donor pockets towards the metal ions [19,20,21,22]. The H_4_L undergoes *enolization* in the presence of metal ions, and coordination to the metal ions takes place via deprotonation of all four acidic hydrogens. In the case of **1**, the ligand coordinates simultaneously with two Cu(II) ions into its two chelate pockets and also binds the third Cu(II) ions by two amide nitrogen donors. In complex **2,** the third metal coordination is absent, suggesting a higher flexibility in the coordination behavior of Cu(II) over oxidovanadium(V). A few binuclear Cu(II) complexes of the same ligand, which were reported (without X-ray crystal structures) earlier, are different in nuclearities and coordination behavior of the ligand [21,22]. Moreover, the authors used Cu(OAc)_2_·H_2_O or Cu(NO_3_)_2_· 2.5H_2_O to synthesize such Cu(II) complexes, whereas in our case, CuCl_2_·2H_2_O was used, affording a hexa-nuclear Cu(II) complex.

Structural characterization of **1** and **2** have been carried out by elemental analysis, FT-IR spectroscopy, ESI-MS, and single crystal X-ray diffraction techniques. 

### 2.2. General Description of the Crystal Structure

X-ray-quality good crystals of compound **1** were obtained from DMF–methanol upon slow evaporation at room temperature. The crystallographic data and selected dimensions of **1** are presented in Table 1 and Table 2. 

**Figure 1:**
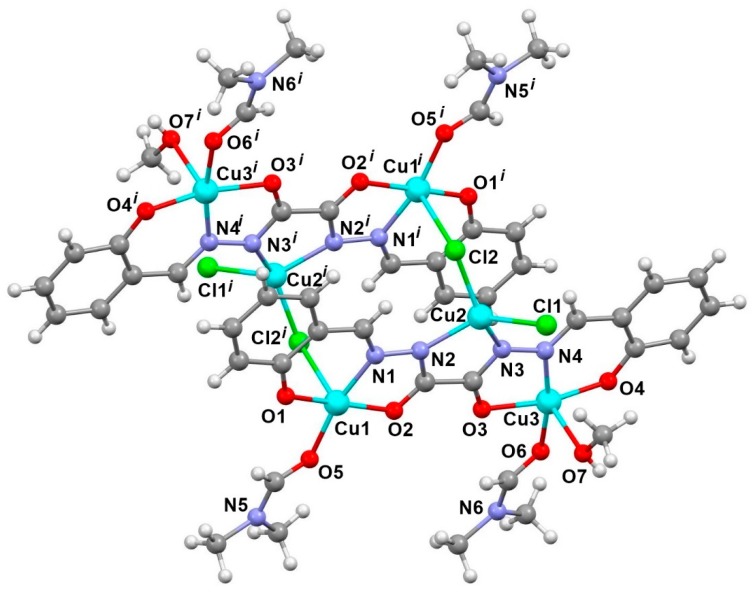
Molecular structural representation of complex **1** with atom labelling scheme. Symmetry codes to generate equivalent atoms: i) -x,1-y,1-z.

**Figure 2:**
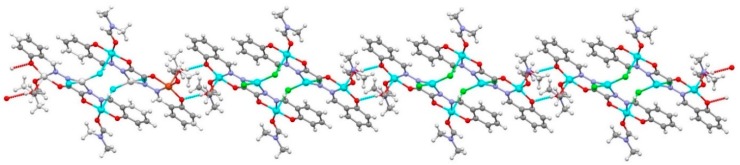
Hydrogen-bonded 1D network of **1**, viewed along the crystallographic *b* axis.

The asymmetric unit of **1** contains one ligand L^4^^−^, two chloride ions, one methanol molecule, two DMF molecules, and three Cu(II) ions (Figure 1). The Schiff base ligand is in the tetra-anionic *enol* form, and the symmetry expansion reveals a hexa-nuclear Cu(II) structure with a metal-to-ligand ratio of 3:1. Two Cu(II) occupy in two dinegative tridentate chelate pockets of the ligand (L^4^^−^) via the O_enolate_, the N_imine_, and the O_phenolate_ atoms. The remaining two N_imine_ atoms of the ligand coordinate to a third Cu(II). Three Cu(II) cations have three different geometries. Both Cu1 and Cu3 exhibit square pyramidal geometry (Cu1, τ5 of 0.09 and Cu3, τ5 of 0.04), and Cu2 exhibits distorted tetrahedral geometry. Cu1 is 0.118 Å above towards the apical Cl2 from the basal plane made from O1, N1, and O2 of the ligand and O5 of the coordinated DMF molecule. This Cl2 bridges to another symmetry related Cu1 of the molecule. Cu3 is 0.148 Å above towards the apical O7 of the methanol molecule from the basal plane made from O3, N4, and O4 of the ligand and O6 of the coordinated DMF molecule. The Cu···Cu distances via azine bridges are 4.752 Å (Cu1···Cu2) and 4.801 Å (Cu2···Cu3), respectively. Intermolecular H-bonding is observed between the phenolate oxygen of the ligands and coordinated methanol molecules, which results in a 1D hydrogen bonded network (Figure 2).

### 2.3. Catalytic Studies

**Scheme 2:**
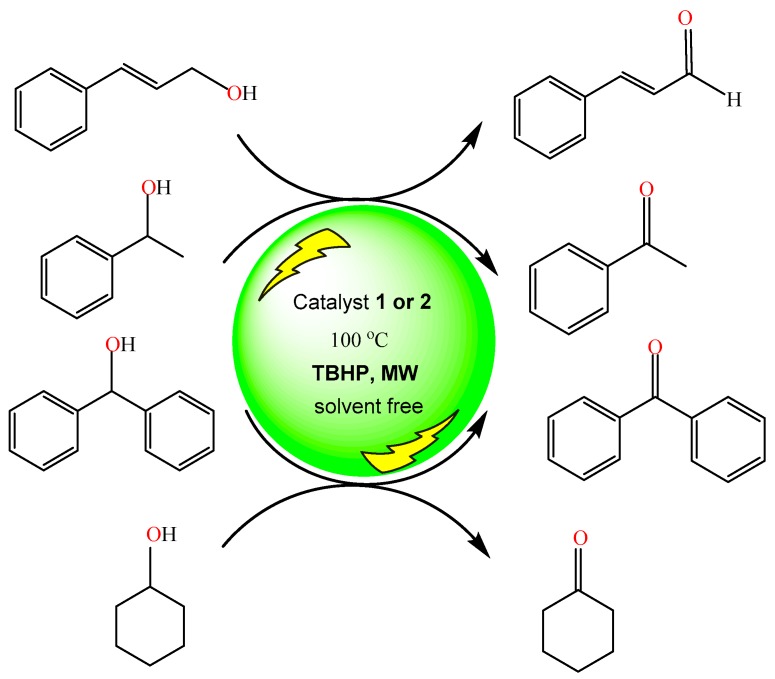
Microwave-assisted neat oxidations of cinnamyl alcohol, 1-phenylethanol, benzhydrol, and cyclohexanol to cinnamaldehyde, acetophenone, benzophenone, and cyclohexanone, respectively, catalyzed by **1** or **2** using aq. *tert*-butyl hydroperoxide (Bu*^t^*OOH, TBHP, 70% aq. solution).

The hexanuclear Cu(II) complex [Cu_3_(μ_2_-1κ*NO^2^*,2κ*NO^2^*-L)(μ-Cl)_2_(Cl)(MeOH)(DMF)_2_]_2_ (**1**) and the dinuclear oxidovanadium(V) complex [{VO(OEt)(EtOH)}_2_(1κ*NO^2^*,2κ*NO^2^*-L)]·2H_2_O (**2**) were tested as homogeneous catalyst precursors for the neat oxidation of primary or secondary benzyl alcohols (cinnamyl alcohol, 1-phenylethanol, benzhydrol) and a secondary aliphatic alcohol (cyclohexanol) to the corresponding carbonyl compounds (aldehydes for primary alcohols and ketones for secondary alcohols; see Scheme 2) under low power (5–10 W) MW irradiation using aqueous *tert*-butyl hydroperoxide (TBHP) as oxidizing agent without any added solvent. Complete (100%) selectivity to the formation of cinnamaldehyde, acetophenone, benzophenone, and cyclohexanone from the corresponding cinnamyl alcohol, 1-phenylethanol, benzhydrol, and cyclohexanol, respectively, was achieved (no traces of byproducts were detected by GC-MS analysis of the final reaction mixtures). 

**Figure 3:**
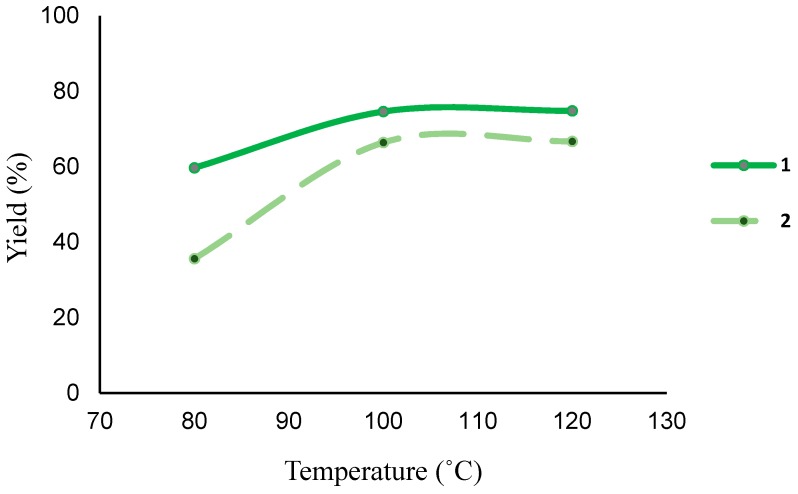
Dependence on the temperature for neat microwave (MW)-assisted oxidation of 1-phenylethanol using **1** or **2** as catalyst precursors. Reaction conditions: 5 mmol of substrate, 10 μmol (0.2 mol% vs. substrate) of **1** or **2**, 10 mmol of TBHP (2 eq., 70% in H_2_O), MW irradiation (5–10 W), 30 min reaction time.

As the model substrate, 1-phenylethanol was chosen for the optimization of the oxidation conditions, starting by building time and temperature profiles. It was observed that by raising the temperature from 80 °C to 100 °C, the catalytic performances of **1** and **2** were enhanced. However, additional increase from 100 °C to 120 °C led to almost the same product yields. Complex **1**, after 30 min under MW irradiation at 100 °C (Figure 3), exhibited the highest activity with a TON (Turnover number = mol of product per mol of catalyst precursor) value of 373, corresponding to a 74.6 % yield of acetophenone (entry 2, Table 3). Under the same reaction conditions, complex **2** yielded 66.4% of acetophenone with a TON value of 332 (entry 23, Table 3). 

**Figure 4:**
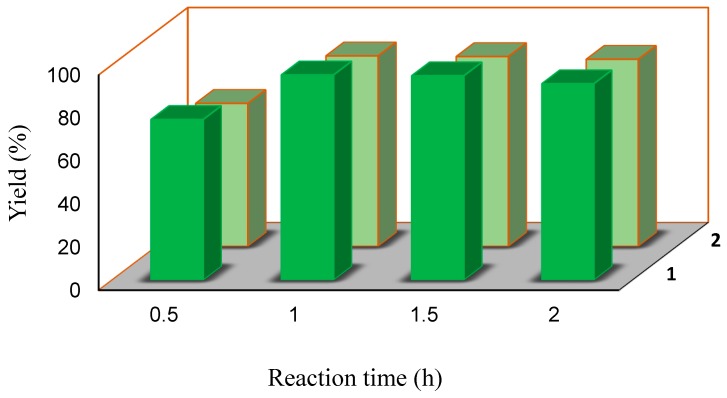
Acetophenone yielded from neat MW-assisted peroxidative oxidation of 1-phenylethanol at different reaction times using **1** or **2** as catalyst precursors. Reaction conditions: 5 mmol of substrate, 10 μmol (0.2 mol% vs. substrate) of **1** or **2**, 10 mmol of TBHP (2 eq., 70% in H_2_O), 100 °C, MW irradiation (5–10 W).

MW irradiation for 1 h at 100 °C was found to be the optimum catalytic condition for yielding acetophenone, achieving the highest yields: 95.3% and 88.5% in the presence of **1** and **2**, respectively (Table 3, entries 4 and 25). For longer reaction times, a slight decrease in the ketone yields were found, conceivably due to overoxidation (Figure 4). 

The heating technique in an oil bath was also applied to catalytic systems **1** and **2** under the above optimized reaction conditions (time and temperature), in the absence of any added solvent, to compare the product yields with those obtained by MW irradiation. Using conventional heating, 76.6% and 67.8% of acetophenone (Table 3, entries 7 and 28) was achieved in the presence of **1** and **2**, respectively, after 1 h at 100 °C, which is significantly lower than that obtained using MW irradiation (95.3% and 88.5% for **1** and **2**, respectively). Moreover, to attain 96.8% of acetophenone in the presence of catalyst **1**, 6 h of oil bath heating at 100 °C was required (Table 3, entry 8); that is, MW irradiation reduces by 1/6^th^ the reaction time required to achieve similar yields to those obtained using a conventional heating mode. These results support the credibility of microwave irradiation as an improved alternative energy technique for energy demanding processes.

**Figure 5:**
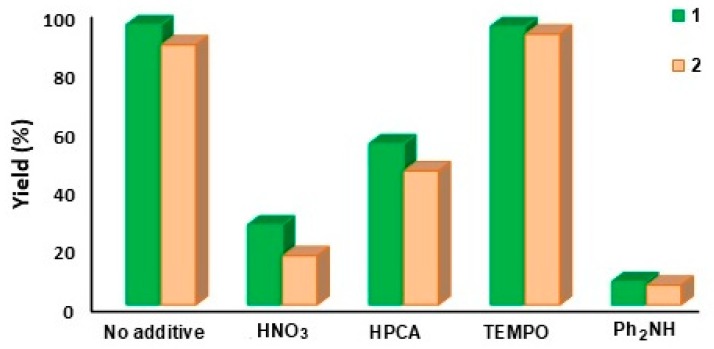
Influence of different additives on the yield of acetophenone obtained from MW-assisted neat peroxidative oxidation of 1-phenylethanol in the presence of catalyst precursor **1**or **2**. Reaction conditions: 5 mmol of substrate, 10 μmol (0.2 mol% vs. substrate) of **1** or **2**, 10 mmol of TBHP (2 eq., 70% in H_2_O), additives [n(additive)/n(catalyst) = 25], 100 °C, MW irradiation (5–10 W).

The effect of different additives on the catalytic ability of complexes **1** and **2** was explored. Nitric acid (HNO_3_) and 2-pyrazinecarboxylic acid (HPCA), usually reported oxidation promotors [1,17,18], as well as 2,2,6,6-tetramethylpiperidine-1-oxyl radical (TEMPO) and diphenylamine (Ph_2_NH), were chosen to test the performances of **1** and **2** for the neat microwave-assisted peroxidative oxidation of 1-phenylethanol at the above optimized conditions (Figure 5). In the presence of HNO_3_, the yields of acetophenone decreased dramatically from 95.3% to 27.2% and 88.5% to 16.7% for **1** and **2**, respectively (Table 3, entries 9 and 29). An inhibiting effect was also observed in the presence of the heteroaromatic acid HPCA (pyrazine-2-carboxylic acid), leading to a decrease of acetophenone yields to 54.8% and 45.6%, for **1** and **2**, respectively (entries 10 and 30 of Table 3, respectively). These results suggest that the acid could break the chloro bridges, causing complexes to loss their structure and inherent reactivity. 

On the contrary, a favorable effect on the acetophenone yield was observed by addition of TEMPO (2,2,6,6-tetramethylpiperidin-1-yl)oxyl) to the reaction mixture in the presence of complex **2,** whereas no effect was detected in the presence of complex **1** (Table 3, entries 31 and 11). The yield of acetophenone was enhanced from 88.5% to 92.1%, the highest yield towards selective oxygenation of 1-phenylethanol being obtained by V(V) complex **2** [TON (TOF = Turnover frequency, mol of product per mol of catalyst precursor per time) values of 461 (461)]. Cu(II) complex **1** led to a maximum yield of 95.3% acetophenone in the absence of any additives, which is higher than the yield previously observed in the presence of the Cu(II) complex [Cu((k*NN*’*O*-HL)(H_2_O)_2_] (H_2_L = *N*-acetylpyrazine-2-carbohydrazide), which required the promotor TEMPO to lead to a 91.3% yield of acetophenone [32]. 

The catalytic activities of **1** and **2** were strongly inhibited for the reactions carried out in the presence of the radical scavenger Ph_2_NH [36] (Table 3, entries 12 and 32). 

**Figure 6:**
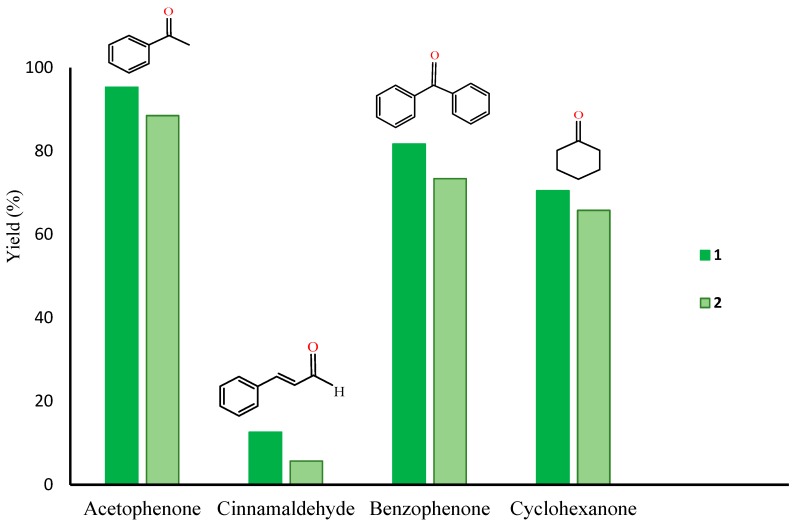
Yield analysis of MW-assisted neat peroxidative oxidation of 1-phenylethanol, cinnamyl alcohol, benzhydrol, and cyclohexanol in the presence of catalyst precursors **1** or **2**. Reaction conditions: 5 mmol of substrate, 10 μmol (0.2 mol% vs. substrate) of **1** or **2**, 10 mmol of TBHP (2 eq., 70% in H_2_O), 100 °C, 1 h, MW irradiation (5–10 W).

The selective catalytic oxidations of cinnamyl alcohol, benzhydrol, and cyclohexanol were tested under the optimized reaction conditions found for 1-phenylethanol. Lower yields of the corresponding ketones or aldehyde were achieved when compared with the obtained values for acetophenone (Figure 6). Moreover, whereas in presence of complex **1**, TEMPO influence was not detected for the oxidation of cinnamyl alcohol, benzhydrol, or cyclohexanol after 1 h at 100 °C under MW irradiation (see Table 3, entries 13, 16, and 19, respectively), TEMPO had a slightly promoting effect when **2** was used, leading to increases of 5.7% to 7.2% for cinnamaldehyde, 73.4% to 79.8% for benzophenone, and 65.8% to 67.9% for cyclohexanone (entries 33–38, Table 3). 

As depicted in Figure 5, the addition of diphenylamine to the reaction mixture led to a significant yield drop compared to the reactions carried out under the same conditions but in the absence of such an oxygen radical trap. This suggests that oxygen radicals generated during the reaction are immediately trapped by the radical scavenger. The proposed mechanism is summed up in Equations (1)–(6), where M is the catalyst metal center. The mechanism may involve the coordination of the alcohol followed by metal-centered dehydrogenation and oxidation of the alcohol through hydrogen abstraction or one-electron oxidation processes [37,38].

M^n+^ + ^t^BuOOH → M^(n+1)+^ −OH + ^t^BuO^●^ (1)

M^(n+1)+^ + ^t^BuOOH → M^n+^ + ^t^BuOO^●^ + H^+^ (2)

M^(n+1)+^ −OH + ^t^BuOOH → M^(n+1)+^ −OO–^t^Bu + H_2_O (3)

^t^BuO^●^ + R_2_CHOH → ^t^BuOH + R_2_C^●^–OH (4)

^t^BuOO^●^ + R_2_CHOH → ^t^BuOOH + R_2_C^●^–OH (5)

M^(n+1)+^ −OO–^t^Bu + R_2_C^●^–OH → R_2_C=O + ^t^BuOOH + M^n+^ (6)

For comparative purposes, the salts used in the synthesis of complexes **1** and **2** were also tested (Table 3, entries 39–46) and yielded up to a maximum of 4.9% of oxidized products. This suggests that the aroylhydrazone ligands have a relevant role in the coordination sphere of Cu and V and therefore, in their catalytic activity. 

In terms of yield and TON values, the Cu(II) complex **1** acts as a more efficient catalyst precursor (highest yield of 95.3% for acetophenone) than V(V) complex **2** (acetophenone maximum yield of 88.5%) under solvent-free and promoter-free conditions. In addition, this new aroylhydrazone Cu(II) complex exhibits higher activity under milder reaction conditions (at lower temperature and in promotor-free conditions) than required for aroylhydrazone Cu(II) analogues [7,31], which constitutes an advantage towards the development of sustainable alcohol oxidation processes. 

To determine the isolated yield for the 1-phenyl ethanol substrate (oxidized in the presence of complex **1**), column chromatography was performed using a mixture of ethyl acetate and n-hexane as eluent, and the purity of the obtained acetophenone was verified by ^1^H NMR (Figure S1). The catalytic reaction was performed with 10 mmol of 1-phenyl ethanol under the optimized oxidation conditions using **1** as catalyst precursor. An 84.1% isolated yield of acetophenone was obtained. This value is almost 11% lower than the yield obtained by GC-MS. The difference in yields may be attributed to some loss of product during the column chromatography process. 

The recyclability of both catalyst precursors was impaired by their low stability under the oxidation conditions, i.e., after the first cycle, the recovery of complex **1** or **2** from the reaction mixture was not possible. In view of the good catalytic performance of our compounds for the oxidation of alcohols, the improvement of their stability upon anchorage on an inert support (e.g., carbon materials or zeolites) is envisaged. 

In order to detect the reactive species, we have analyzed the reaction solution by ESI-MS without adding any substrate. A metal-organoperoxido species, [Cu_3_(L^4−^)(DMF)_2_(OO)(OOH)]^−^ (*m/z* = 724), was observed when the oxidation was undertaken in the absence of the alcohol.

## 3. Materials and Methods 

The synthesis of the pro-ligand and its Cu(II) and V(V) complexes for this study was performed in open air. Reagents and solvents were used as received commercially, without further purification or drying. CuCl_2_·2H_2_O and VO(acac)_2_ were used as metal precursors for the synthesis of the complexes. Elemental analyses (C, H, and N) were carried out by the Microanalytical Service of the Instituto Superior Técnico. A Bruker Vertex 70 instrument (Bruker Corporation, Ettlingen, Germany) was used for infrared spectra (4000–400 cm^−1^) analysis in KBr pellets; wavenumbers are in cm^−1^. The ^1^H NMR spectrum of the ligand was recorded on a Bruker Avance II + 400.13 MHz (UltraShield^TM^ Magnet) spectrometer at room temperature. The internal reference was tetramethyl silane, and the chemical shifts are reported in ppm in the ^1^H NMR spectrum. Mass spectra were recorded in a Varian 500-MS LC Ion Trap Mass Spectrometer (Agilent Technologies, Amstelveen, The Netherlands) equipped with an electrospray (ESI) ion source. For electrospray ionization, the drying gas and flow rate were optimized according to the particular sample with 35 p.s.i. nebulizer pressure. Scanning was carried out from *m/z* 100 to 1200 in methanol solution. The compounds were observed in the positive mode (capillary voltage = 80–105 V).

### 3.1. Syntheses of the Pro-Ligand H_4_L 

The aroylhydrazone Schiff base *N′^1^,N′^2^*-bis(2-hydroxybenzylidene)oxalohydrazide (H_4_L) was prepared according to the literature [20] upon condensation of the oxalyldihydrazide with salicylaldehyde. 

Yield: 84.0%. Anal. calc. for C_16_H_14_N_4_O_4_: C, 58.89; H, 4.32; N, 17.17. Found: C, 58.83; H, 4.29; N, 17.15. IR (KBr pellet, cm^−^^1^): 3306 ν(OH), 3038 ν(NH), 1668 ν(C=O), 1036 ν(N–N). ^1^H NMR (DMSO-*d*_6_, *δ*): 12.64 (s, 2H, NH), 11.02 (s, 2H,OH), 6.76–7.68 (m, 8H, C_6_H_4_), 8.82(s, 2H, NH), 1.92 (s, 3H,CH_3_).

### 3.2. Synthesis of [Cu_3_(μ_2_-1κNO^2^,2κNO^2^-L)(μ-Cl)_2_(Cl)(MeOH)(DMF)_2_]_2_ (**1**)

The pro-ligand H_4_L (0.330 g, 1.01 mmol) was dissolved in 10 mL of DMF and added to the methanolic (20 mL) solution of the copper salt CuCl_2_**·**2H_2_O (0.680 g, 4.00 mmol). The reaction mixture was stirred for 30 min at 50 °C. The resultant dark green solution was filtered, and the filtrate was kept in open air for crystallization. Nice green single crystals were isolated after ca. 5 days, suitable for X-ray diffraction analysis. 

Yield: 74%. Anal. Calcd for C_46_H_56_Cl_4_Cu_6_N_12_O_14_ (**1**): C, 36.25; H, 3.70; N, 11.03. Found: C, 36.17; H, 3.66; N, 11.01. IR (KBr pellet, cm^−^^1^): 3268 ν(OH), 1579 ν(C=N), 1247 ν(C–O) enolic and 1058 ν(N–N). ESI-MS(+): *m/z* 763 [½(M)+H]^+^ (100%).

### 3.3. Synthesis of [{VO(OEt)(EtOH)}_2_(1κNO^2^,2κNO^2^-L)]·2H_2_O (**2**)

The dinuclear oxidovanadium(V) complex **2** was synthesized as described in the literature [20]. 

Yield 72 %. Anal. Calcd for C_24_H_36_ N_4_O_12_V_2_: C, 42.74; H, 5.38; N, 8.31. Found: C, 42.64; H, 5.33; N, 8.27. IR (KBr pellet, cm^−^^1^): 3230 ν(OH), 1606 ν(C=N), 1036 ν(N–N), 976 ν(V=O). ESI-MS(+): *m/z* 548 [M-2(EtOH+H_2_O)+H]^+^ (100 %).

### 3.4. X-ray Measurements

Good quality single crystal of complex **1** (CCDC 1993276) suitable for X-ray diffraction was immersed in cryo-oil, mounted in Nylon loops, and measured at a temperature of 297 K. Intensity data were collected using a Bruker AXS PHOTON 100 diffractometer with graphite monochromated Mo-Kα (λ 0.71073) radiation. Data collections were recorded using omega scans of 0.5° per frame and full spheres of data were obtained. Cell parameters were retrieved using Bruker SMART [39] software and the data were refined using Bruker SAINT [39] on all the observed reflections. Absorption corrections were applied using SADABS [39]. Structures were solved by direct methods by using SIR97 [40] and refined with SHELXL2014 [41]. Calculations were performed using WinGX v2014.1 [42]. The H-atoms bonded to carbon were included in the model at geometrically calculated positions and refined using a riding model. U_iso_(H) were defined as 1.2U_eq_ of the parent carbon atoms for aromatic residues and 1.5U_eq_ for the methyl groups. The other hydrogen atoms (N–H) were in calculated positions as aromatics located in the difference Fourier synthesis and refined. Least-square refinements were employed with anisotropic thermal motion parameters for all the non-hydrogen atoms and isotropic parameters for the remaining atoms. 

### 3.5. Catalytic Studies 

The catalytic tests were performed under MW irradiation in a focused microwave Anton Paar Monowave 300 discover reactor (5–10 W) using a 10 mL capacity reaction tube with a 13 mm internal diameter, fitted with a rotational system and an IR (Infrared) temperature detector. Gas chromatography (GC) measurements were carried out using a FISONS Instruments GC 8000 series gas chromatograph with a DB-624 (J&W) capillary column (flame ionization detector or FID detector) and the Jasco-Borwin v.1.50 software. The temperature of injection was 240 ˚C. The initial temperature was maintained at 120 ˚C for 1 min then raised 10 ˚C/min to 200 ˚C and held at this temperature for 1 min. Helium was used as the carrier gas. GC-MS analyses were performed using a Perkin-Elmer Clarus 600 C instrument (He as the carrier gas). The ionization voltage was 70 eV. Gas chromatography was conducted in the temperature-programming mode using a SGE BPX5 column (30 m × 0.25 mm × 0.25 μm). Reaction products were identified by comparison of their retention times with known reference compounds and by comparing their mass spectra to fragmentation patterns obtained from the NIST spectral library stored in the computer software of the mass spectrometer.

#### Typical Procedures for the Catalytic Oxidation of Alcohols and Product Analysis

Oxidation reactions of the alcohols were carried out in sealed cylindrical Pyrex tubes under focused MW irradiation as follows: the alcohol substrate (5 mmol), catalyst precursor **1–3** (10 μmol, 0.2 mol% vs. substrate), and a 70% aqueous solution of *^t^*BuOOH (10 mmol) were introduced in the tube. This was then placed in the microwave reactor, and the system was stirred and irradiated (5–10 W) for 0.5–2 h at 80–120 ˚C. After the reaction, the mixture was allowed to cool down to room temperature. A total of 300 μL of benzaldehyde (internal standard) and 5 mL of NCMe (to extract the substrate and the organic products from the reaction mixture) were added. The obtained mixture was stirred for 10 min, and then a sample (1 μL) was taken from the organic phase and analyzed by GC using the internal standard method. 

## 4. Conclusions

The catalytic ability of a newly synthesized hexanuclear Cu(II) complex [Cu_3_(μ_2_-1κ*NO^2^*,2κ*NO^2^*-L)(μ-Cl)_2_(Cl)(MeOH)(DMF)_2_]_2_ (**1**) and a dinuclear oxidovanadium(V) [{VO(OEt)(EtOH)}_2_(1κ*NO^2^*,2κ*NO^2^*-L)]·2H_2_O (**2**) complex for the microwave-assisted neat peroxidative oxidation of alcohols was successfully explored.

Four different types of alcohol substrates were used as model substrates for this study, and the catalytic performances of **1** and **2** were compared. Both catalyst precursors were efficient in terms of yields and selectivities. However, the new aroylhydrazone Cu(II) complex exhibited higher activity under milder reaction conditions (at lower temperature and in promotor-free conditions) than the dinuclear oxidovanadium(V) complex or aroylhydrazone Cu(II) analogues previously reported, which constitutes an advantage towards the development of sustainable alcohol oxidation processes. Nevertheless, complex **2** exhibited the highest catalytic activity per metal center (in terms of yield and TON values). Thus, our microwave-assisted neat catalytic Cu-system appears to be useful for energy saving and improvement of the sustainability of the catalytic oxidations. Further research should thus be performed to enhance the stability of the catalyst precursors (e.g., by anchorage on inert supports such as carbon materials or zeolites) under the oxidation conditions to allow them to be re-used.

## Figures and Tables

**Table 1:** Crystal data and structure refinement details for complex **1**.

Parameters	1
Empirical formula	C_23_H_28_Cl_2_Cu_3_N_6_O_7_
Formula weight	762.03
Crystal system	Triclinic
Space group	*P*¯1
Temperature/K	296 (2)
*a*/Å	7.7742 (9)
*b*/Å	11.9744 (12)
*c*/Å	17.287 (2)
α/°	104.505 (6)
*β/*°	98.430 (6)
γ/°	106.176 (4)
*V* (Å^3^)	1455.2 (3)
*Z*	2
D_calc_ (g cm^−3^)	1.739
*μ* (Mo K*α*) (mm^−1^)	2.41
Rfls. collected/unique/observed	28371/7251/4657
*R*_int_	0.082
Final *R*1 *^a^*, *wR*2 *^b^* (*I* ≥ 2*σ*)	0.044, 0.087
Goodness-of-fit on *F*^2^	1.01

*^a^* R *=* Σ||*F_o_*|–|*F_c_*||/Σ|*F_o_*|; ^*b*^ wR(F^2^) = [Σw(|F_o_|^2^ – |F_c_|^2^)^2^/Σw|F_o_|^4^]^½^.

**Table 2:** Selected bond distances (Å) and angles (°) in complex **1**.

Cu2—N3	1.978 (3)	Cu3—O6	1.983 (2)
Cu2—N2	2.009 (3)	Cu3—O7	2.321 (3)
Cu2—Cl1	2.2258 (10)	Cu1—O1	1.887 (2)
Cu2—Cl2	2.2307 (9)	Cu1—N1	1.937 (3)
Cu3—O4	1.904 (2)	Cu1—O5	1.953 (2)
Cu3—N4	1.933 (3)	Cu1—O2	1.962 (2)
Cu3—O3	1.977 (2)		
N3—Cu2—N2	80.89 (10)	O3—Cu3—O6	90.12 (9)
N3—Cu2—Cl1	97.27 (8)	O4—Cu3—O7	92.69 (11)
N2—Cu2—Cl1	142.23 (9)	N4—Cu3—O7	93.64 (11)
N3—Cu2—Cl2	147.92 (9)	O3—Cu3—O7	98.84 (10)
N2—Cu2—Cl2	103.72 (8)	O6—Cu3—O7	92.74 (11)
Cl1—Cu2—Cl2	97.50 (4)	O1—Cu1—N1	94.45 (10)
O4—Cu3—N4	93.33 (10)	O1—Cu1—O5	93.38 (10)
O4—Cu3—O3	167.56 (11)	N1—Cu1—O5	168.00 (11)
N4—Cu3—O3	81.34 (10)	O1—Cu1—O2	173.56 (11)
O4—Cu3—O6	94.00 (10)	N1—Cu1—O2	81.41 (10)
N4—Cu3—O6	170.04 (11)	O5—Cu1—O2	89.97 (10)

**Table 3:** Selected catalytic results *^a^* for the selective neat oxidation of alcohols with TBHP (70% aq.) under MW irradiation and using complex **1** or **2** as catalyst precursor.

Entry	Catalyst	Substrate	Temperature (°C)	Reaction Time (h)	Additive	Yield (%) *^b^*	TON (TOF (h^−1^)) *^c^*
1	**1**	1-phenyl ethanol	80	0.5	-	59.7	299 (598)
2			100	0.5	-	74.6	373 (746)
3			120	0.5	-	74.8	374 (748)
4			100	1.0	-	95.3	476 (476)
5			100	1.5	-	94.9	475 (317)
6			100	2.0	-	91.2	327 (164)
7 *^d^*			100	1	_-_	76.6	383 (383)
8 *^d^*			100	6		96.8	484 (81)
9 *^e^*			100	1	HNO_3_	27.2	136 (136)
10 *^f^*			100	1	HPCA	54.8	274 (274)
11 *^g^*			100	1	TEMPO	94.7	474 (474)
12 *^h^*			100	1	Ph_2_NH	7.9	40 (40)
13		cinnamyl alcohol	100	1	-	12.6	63 (63)
14 *^g^*			100	1	TEMPO	12.0	60 (60)
15 *^h^*			100	1	Ph_2_NH	1.6	8 (8)
16		benzhydrol	100	1	-	81.7	409 (409)
17 *^g^*			100	1	TEMPO	80.9	405 (405)
18 *^h^*			100	1	Ph_2_NH	3.2	16 (16)
19	cyclohexanol		100	1	-	70.5	353 (353)
20 *^g^*			100	1	TEMPO	71.0	355 (355)
21*^h^*			100	1	Ph_2_NH	3.4	178 (356)
22	**2**	1-phenyl ethanol	80	0.5	-	35.6	178 (356)
23			100	0.5	-	66.4	332 (664)
24			120	0.5	-	66.7	326 (652)
25			100	1.0	-	88.5	443 (443)
26			100	1.5	-	88.2	441 (294)
27			100	2.0	-	86.9	435 (218)
28 *^d^*			100	1.0	-	67.8	339 (339)
29 *^e^*			100	1.0	HNO_3_	16.7	84 (84)
30 *^f^*			100	1.0	HPCA	45.6	228 (228)
31 *^g^*			100	1.0	TEMPO	92.1	461 (461)
32 *^h^*			100	1.0	Ph_2_NH	6.7	34 (34)
33		cinnamyl alcohol	100	1.0	-	5.7	133 (133)
34 *^g^*			100	1.0	TEMPO	7.2	36 (36)
35		benzhydrol	100	1.0	-	73.4	367 (367)
36 *^g^*			100	1.0	TEMPO	79.8	399 (399)
37		cyclohexanol	100	1.0	-	65.8	329 (329)
38 *^g^*			100	1.0	TEMPO	67.9	340 (340)
39	CuCl_2_·2H_2_O	1-phenyl ethanol	100	1.0	-	6.2	31 (31)
40		cinnamyl alcohol	100	1.0	-	1.7	9 (9)
41		benzhydrol	100	1.0	-	4.4	22 (22)
42		cyclohexanol	100	1.0	-	3.5	18 (18)
43	VO(acac)_2_	1-phenyl ethanol	100	1.0	-	4.9	25 (25)
44		cinnamyl alcohol	100	1.0	-	1.1	6 (6)
45		benzhydrol	100	1.0	-	3.6	18 (18)
46		cyclohexanol	100	1.0	-	2.8	14 (14)

*^a^* Reaction conditions: 5 mmol of substrate, 10 μmol (0.2 mol% vs. substrate) of catalyst precursor **1** or **2**, 10 mmol of TBHP (2 eq., 70% in H_2_O), MW irradiation (5–10 W). *^b^* Moles of ketone product per 100 moles of alcohol. *^c^* Turnover number = number of moles of product per mol of catalyst precursor; TOF = TON per hour (values in brackets). *^d^* Conventional heating. *^e^* n(HNO_3_)/n(catalyst) = 25. *^f^* n(HPCA)/n(catalyst) = 25. *^g^* n(TEMPO)/n(catalyst) = 25. *^h^* n(Ph_2_NH)/n(catalyst) = 25.

## Supplementary Materials

Supplementary data to this article can be found online at https://www.mdpi.com/1422-0067/21/8/2832/s1. CCDC 1993276 for **1** contains the supplementary crystallographic data for this paper. This data can be obtained free of charge from The Cambridge Crystallographic Data Centre via www.ccdc.cam.ac.uk/data_request/cif.
