# Supplementary figures and images for: Aroylhydrazone Schiff Base Derived Cu(II) and V(V) Complexes: Efficient Catalysts towards Neat Microwave-Assisted Oxidation of Alcohols
Source: Int J Mol Sci. 2020 Apr 18;21(8):2832. doi: 10.3390/ijms21082832 (PMC7215666; doi:10.3390/ijms21082832)

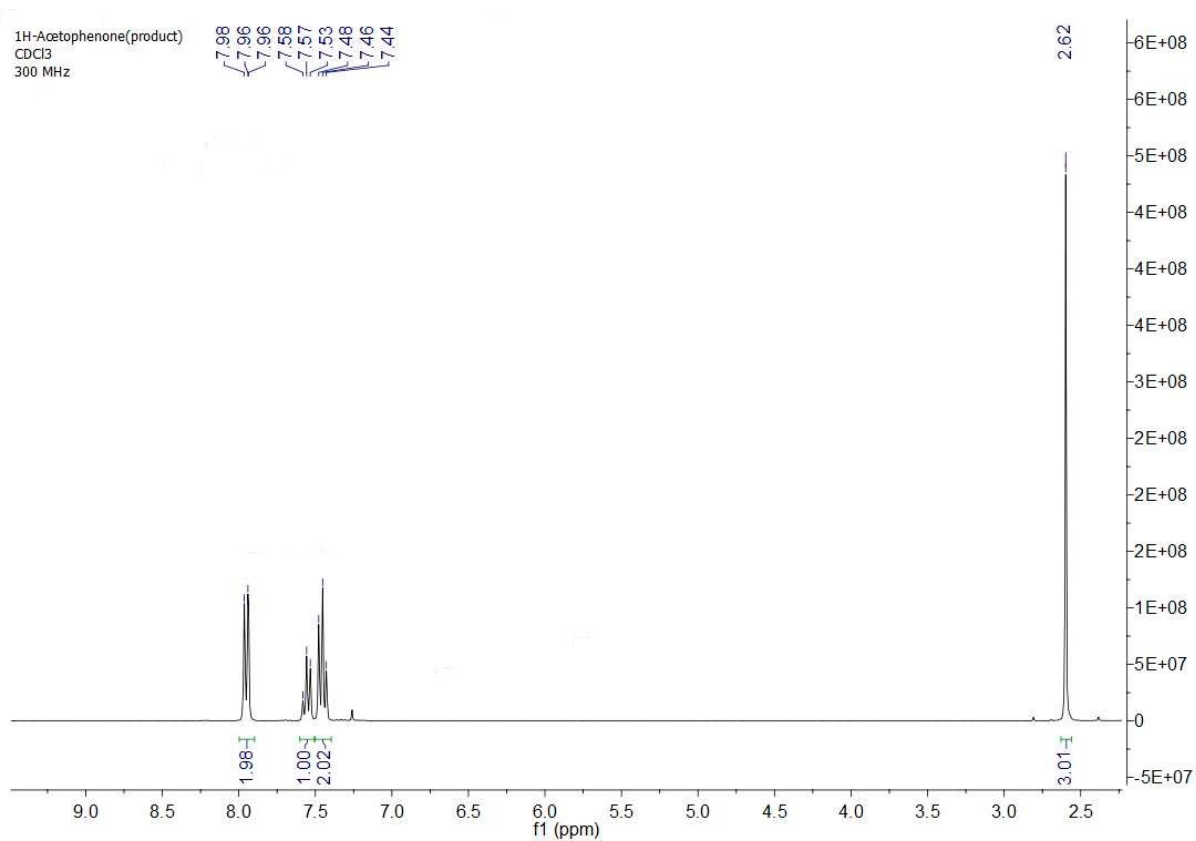

**Figure S1.** <sup>1</sup>H NMR spectrum of acetophenone (obtained from column chromatography).

Supplement: Supplementary file 1 [file ijms-21-02832-s001.pdf]
